# Functional cerebral reorganization: a signature of expertise? Reexamining Guida, Gobet, Tardieu, and Nicolas' (2012) two-stage framework

**DOI:** 10.3389/fnhum.2013.00590

**Published:** 2013-09-20

**Authors:** Alessandro Guida, Fernand Gobet, Serge Nicolas

**Affiliations:** ^1^Département de Psychologie, Centre de Recherche en Psychologie, Cognition et Communication, Université Rennes 2Rennes, France; ^2^Department of Psychological Sciences, University of LiverpoolLiverpool, UK; ^3^Institut de Psychologie, Université Paris DescartesBoulogne Billancourt, France

**Keywords:** expertise, working memory, functional cerebral reorganization, chunks, templates, retrieval structures

In 2012, Guida, Gobet, Tardieu and Nicolas proposed a two-stage framework to explain how cognitive changes due to practice could shape experts' brain physiologically and thus explain neuroimaging data of expertise acquisition. In this paper, after presenting the motivations for such a framework and the framework itself, we examine the idea that functional cerebral reorganization (FCR) could be used as a signature for expertise.

## Chunks, templates and retrieval structures

In the mid-nineties, Ericsson and Kintsch (1995) and Gobet and Simon (1996) proposed Long-Term Working Memory theory (LTWMT) and Template Theory (TT), respectively, in order to account for behavioral data in the domain of expertise. These data were difficult to explain with the sole concept of chunk (Chase and Simon, 1973), given the severe limitations of working memory (WM) (7 ± 2 for an optimistic estimation, Miller, 1956; but for recent reevaluations, see Cowan, 2001; Gobet and Clarkson, 2004; Mathy and Feldman, 2012). For example, several experiments (e.g., Charness, 1976; Frey and Adesman, 1976; Glanzer et al., 1984) showed that interfering tasks had almost no effect on WM performance or text comprehension with experts. Yet, according to chunking theory (Chase and Simon, 1973), interfering tasks should wipe out the content of WM where information is stored. This led Ericsson and Kintsch (1995) and Gobet and Simon (1996) to suggest that information was not stored in WM as initially proposed, but was rapidly and efficiently transferred in LTM, where the interfering tasks has no effect. Both theories proposed that this was possible only if knowledge structures were built. These structures were called templates with TT and retrieval structures with LTWMT. Even if differences exist between the two theories (e.g., Ericsson and Kintsch, 2000; Gobet, 2000a,b), LTWMT and TT revolve around the same fundamental core idea: Fast and reliable transfer in LTM becomes possible with expertise via knowledge structures, which enables LTM to be used during WM tasks, thus giving the appearance of expanding individuals' WM capacity. These two cognitive theories have been used to explain not only behavioral but also neuroscientific data (e.g., Pesenti et al., 2001; Ericsson, 2003; Campitelli et al., 2007; Bilalić et al., 2010).

## Explaining neuroimaging data in expertise acquisition: A two-stage framework

Recently, the core idea of the two theories has been used by Guida et al. (2012) to bridge together, for the first time, (a) neuroimaging data acquired from novice undergoing practice in WM-related tasks and (b) neuroimaging data acquired from experts in WM-related tasks. The results of the two groups of studies, which belong to two separate domains of research, diverge. Neuroimaging of novices practicing from 2 h up to 5 weeks show mainly a decrease of activation in prefrontal and parietal areas (for similar conclusions, see Kelly and Garavan, 2005; Hill and Schneider, 2006; Buschkuehl et al., 2012). Conversely, neuroimaging studies of experts who are compared to novices are more compatible with FCR, viz., experts and novices use different brain areas and different mental operations to perform similar tasks (for similar conclusions, see Ericsson, 2003). Notwithstanding these divergent findings (brain activation decrease vs. FCR), the core idea behind LTMWT and TT allows bridging these two neuroimaging patterns into a coherent two-stage framework.

## First stage: decrease of activation due to chunk creation and retrieval

When novices start practicing, and if the activity is new, the first important process is chunk creation. While executing their new activity several times, novices will start gradually chunking separate elements together through binding, viz., encoding the relations among stimuli that co-occur (Cohen and Eichenbaum, 1993). Once chunks have been created and thus stored in LTM, chunk can be retrieved and therefore used, allowing encoding multiple elements in WM with one chunk (e.g., “f,” “b,” “i,” can be encoded as one element in WM instead of three), using fewer resources.

**Figure 1:**
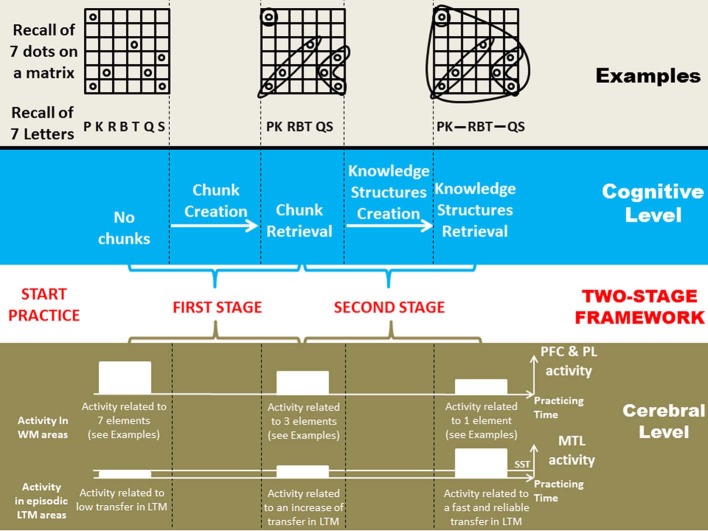
**Schematic representation of the Two-Stage Framework linking the cognitive and cerebral levels in expertise acquisition, through two examples.** The “Examples” section shows the evolution of the effect of knowledge on how items to-be-remembered are processed: at first, items are processed almost separately, later, items are regrouped in chunks, and finally in knowledge structures, which can be viewed as super-chunks that regroup multiple chunks into a high-level pattern. In the “Cerebral Level” section, the representation of brain activity is at an ordinal scale. SST stands for statistical significance threshold; if brain activity is beneath this threshold, it goes undetected. PFC stands for prefrontal cortex, PL for parietal lobe, and MTL for medial temporal lobe. The first MTL activity on the left is almost at the same level than the statistical significance threshold in order to indicate that for novices, brain activity is sometimes detected (see section “Concluding Remarks”). For novices, detection seems to vary according to the kind of experimental paradigm, the parameters and maybe the participants of the experiments. If one considers that the MTL activity is above the statistical significance threshold for novices then functional cerebral reorganization is better suited to describe expertise acquisition; if it is beneath, then functional cerebral redistribution is better suited.

At a physiological level, chunk creation (through binding) and chunk retrieval are two reasons to expect brain activation decrease. First, if the binding process occurs in prefrontal regions (Prabhakaran et al., 2000; Raffone and Wolters, 2001) and in parietal regions (Shafritz et al., 2002; Oakes et al., 2006), less activation should be observed in these regions after a period of training, because as training progresses, fewer chunks will be created. Second, the use of chunks through chunk retrieval makes it possible to encode information in WM with less resources, as elements are grouped. Several researchers have shown that, physiologically, there is a correlation between the number of elements in WM and brain activity in prefrontal and parietal areas^1^ (Todd and Marois, 2004; Vogel and Machizawa, 2004; Cowan, 2011). Therefore, if less WM space is used through chunk retrieval, decrease of brain activity should be expected in prefrontal and parietal WM areas (Figure 1).

## Second stage: functional cerebral reorganization due to knowledge structures creation and retrieval

With practice (e.g., Cowan et al., 2004; Chen and Cowan, 2005) and expertise (e.g., Chase and Simon, 1973; Gobet and Simon, 1996), chunks get larger and more complex, and with years of training they become knowledge structures. For experts, the peculiarity of these structures, when used in their domain of expertise, is to allow rapid and reliable encoding in episodic LTM, even in WM-like conditions (fast presentation times of multiple elements) when usually elements can only be encoded reliably in WM (Figure 1).

In terms of brain activation, at this stage, not only a cerebral activation pattern compatible with WM activities is expected but conjunctly a pattern compatible with episodic LTM, that is, medial temporal lobe (MTL) activations (Gabrieli et al., 1997; Young et al., 1997; Lepage et al., 1998; for reviews, see Squire et al., 2004; Eichenbaum et al., 2007) due to the utilization of knowledge structures. From a longitudinal standpoint, this implies a FCR, which can be defined by two changes occurring with practice: (a) the decrease of brain activity undergirding cognitive processes that are used less with practice (here WM in stage 1), and (b) the emergence of brain activity in new areas supporting new cognitive processes (here episodic LTM in stage 2). Therefore, a FCR involving episodic LTM^2^ is expected (Figure 1). Unfortunately, to our knowledge, nobody has followed the development of expertise in a WM-related task with neuroimaging long enough to test this hypothesis. Instead, what is possible is to compare novices against experts. This was the aim of Guida et al.'s (2012) review, which showed that most of the studies were compatible with FCR involving LTM.

## Functional cerebral reorganization: a signature of expertise?

Given that a link has been established between expertise and FCR, an important question is to know whether FCR could be used as a signature for expertise. A simple way to answer this question is to examine empirically whether the implication “expertise thus FCR” observed by Guida et al. (2012) could be reversed. In other words, when one looks for patterns compatible with FCR—viz. a decrease of brain activity concerning one cognitive process and the emergence of brain activity concerning new cognitive processes—is expertise found? If it is not the case then FCR does not imply expertise.

At first glance, this does not seem to be true. There are multiple examples showing that the simple utilization of different strategies can involve patterns of activation similar to FCR. For instance, the literature of WM-related tasks shows that when different groups of individuals use spontaneously different strategies—verbal strategy vs. visual strategy (Burbaud et al., 2000), or verbal strategy vs. spatial strategy (Glabus et al., 2003)—then completely different patterns of activation are detected. This seems to be true even when the strategies are dictated by the experimenter, as observed by Bernstein et al. (2002) when imposing different encoding strategies in a task of face recognition. These three between-subject studies bring only indirect evidence, but they are confirmed by a within-subject study. When Reichle et al. (2000) asked the same individuals to process a sentence-picture verification task with different strategies (linguistic vs. visual), completely different patterns of activity appeared: there was a decrease of brain activity concerning cognitive processes (e.g., linguistic) and the emergence of brain activity concerning new cognitive processes (e.g., visual). In all these cases, a pattern consistent with FCR is present but no expertise is found. Therefore, the implication “expertise thus FCR” does not seem reversible.

However, when considering precisely FCR involving episodic LTM areas, the picture is different. First, we found only one study (Kondo et al., 2005); secondly, it is the only study where participants were taught how to use knowledge structures. Kondo et al. (2005) asked their novice participants to encode ten object pictures using the method of loci, basing themselves on the visuospatial knowledge of their house. When comparing neuroimaging before and after using the method of loci, they observed a pattern consistent with FCR at retrieval. Hence, if one argues that the method of loci is based on the utilization of expertise (Guida et al., 2009, 2013), the conclusion from Kondo et al. (2005) could be that for FCR involving episodic LTM areas, the implication “expertise thus FCR” can be reversed, making the proposal that FCR is a signature for expertise verisimilar (when involving episodic LTM).

However, when trying to relate expertise and FCR and before one can be conclusive on the link between these two concepts, two elements need to be taken into consideration, functional cerebral redistribution and brain connectivity. These will constitute our concluding remarks.

## Concluding remarks

A very recent growing body of data suggests that in some cases, functional cerebral redistribution could also occur with practice. Both FCR and functional cerebral redistribution involve a combination of increases and decreases in activation (Kelly and Garavan, 2005); however, only FCR necessitates the emergence of new areas with practice. Recent evidence suggests that MTL could also be involved in WM tasks with no practice (e.g., Ranganath and Blumenfeld, 2005; Olson et al., 2006; Lee and Rudebeck, 2010; Campo et al., 2013). The debate is still ongoing and these results are considered artifactual by some, mainly because the tasks used seem more LTM-like than WM-like (Jonides et al., 2008). Squire and Wixted (2011) observed that if WM capacity were not exceeded, MTL was not involved (e.g., Shrager et al., 2008; Jeneson et al., 2012). Nonetheless, these data suggest that in some cases, MTL activation could be expected at the early stages of training. It is plausible that this activation could increase with expertise when knowledge structures are available, which means that this pattern would be better described by functional redistribution than FCR, because this last pattern implies no MTL activation at the initial stage of practice (Figure 1). However, the additional areas of experts are sometimes the same structures than that of novices but on the opposite hemisphere (e.g., Bilalić et al., 2011, 2012), a.k.a. “double take” phenomenon (e.g., Scalf et al., 2007), which complicates sometime the distinction between functional redistribution and FCR. To conclude concerning MTL, more work needs to be done to ascertain its involvement, especially in practice-related studies where this kind of evidence is scarce (but, see Dahlin et al., 2008), therefore, presently, these are only assumptions.

Finally, when considering expertise-related FCR, which constitutes a combined increase and decrease in activation across the brain, it is also crucial to understand how the different brain areas work together in terms of network connectivity. Fundamental in this respect is the idea of “neural context” proposed by McIntosh (1998; also, see Bressler and McIntosh, 2007), according to which the frame of activation (or the neural context of activation) around a determined brain area is at least as important as the activation of that brain area. If one relates this idea to practice, then the consequence is that even if the activation of a region does not change with practice, it can still be crucial, by influencing the increase or decrease of activation in other brain areas (Kelly and Garavan, 2005). The neural context could thus be important for the study of functional reorganization, and its application should be disseminated (Bressler and Menon, 2010).

### Conflict of interest statement

The authors declare that the research was conducted in the absence of any commercial or financial relationships that could be construed as a potential conflict of interest.

## Footnotes

^1^It is particularly the case in the intra-parietal sulcus (e.g., Majerus et al., 2010; Cowan et al., 2011).

^2^FCR, in this article, means, a shift of a way of performing a task to another way, without specifying the new way. FCR involving episodic LTM, in this article, means that the new way of performing the task is through episodic LTM

## References

[B1] Bernstein L. J. Beig S. Siegenthaler A. L. Grady C. L. (2002). The effect of encoding strategy on the neural correlates of memory for faces. Neuropsychologia 40, 86–98 10.1016/S0028-3932(01)00070-7 11595264

[B2] Bilalić M. Kiesel A. Pohl C. Erb M. Grodd W. (2011). It takes two-skilled recognition of objects engages lateral areas in both hemispheres. PLoS ONE 6:e16202 10.1371/journal.pone.0016202 21283683 PMC3025982

[B3] Bilalić M. Langner R. Erb M. Grodd W. (2010). Mechanisms and neural basis of object and pattern recognition – a study with chess experts. J. Exp. Psychol. Gen. 139, 728–742 10.1037/a0020756 21038986

[B4] Bilalić M. Turella L. Campitelli G. Erb M. Grodd W. (2012). Expertise modulates the neural basis of context dependent recognition of objects and their relations. Hum. Brain Mapp. 33, 2728–2740 10.1002/hbm.21396 21998070 PMC6870017

[B5] Bressler S. L. McIntosh A. R. (2007). The role of neural context in large-scale neurocognitive network operations, in Handbook of Brain Connectivity, eds Jirsa V. K. McIntosh A. R. (New York, NY: Springer), 403–419 10.1007/978-3-540-71512-2_14

[B6] Bressler S. L. Menon V. (2010). Large-scale brain networks in cognition: emerging methods and principles. Trends Cogn. Sci. 14, 277–290 10.1016/j.tics.2010.04.004 20493761

[B7] Burbaud P. Camus O. Guehl D. Bioulac B. Caille J. M. Allard M. (2000). Influence of cognitive strategies on the pattern of cortical activation during mental subtraction. A functional imaging study in human subjects. Neurosci. Lett. 287, 76–80 10.1016/S0304-3940(00)01099-5 10841995

[B8] Buschkuehl M. Jaeggi S. M. Jonides J. (2012). Neuronal effects following working memory training. Dev. Cogn. Neurosci. 2 Suppl. 1, S167–S179 10.1016/j.dcn.2011.10.001 22682905 PMC6987667

[B9] Campitelli G. Gobet F. Head K. Buckley M. Parker A. (2007). Brain localisation of memory chunks in chessplayers. Int. J. Neurosci. 117, 1641–1659 10.1080/00207450601041955 17987468

[B10] Campo P. Garrido M. I. Moran R. J. García-Morales I. Poch C. (2013). Network reconfiguration and working memory impairment in mesial temporal lobe epilepsy. Neuroimage 72, 48–54 10.1016/j.neuroimage.2013.01.036 23370058 PMC3610031

[B11] Charness N. (1976). Memory for chess positions: resistance to interference. J. Exp. Psychol. Learn Mem. Cogn. 2, 641–653 10.1037/0278-7393.2.6.641

[B13] Chase W. G. Simon H. A. (1973). Perception in chess. Cogn. Psychol. 4, 55–81 10.1016/0010-0285(73)90004-2

[B14] Chen Z. Cowan N. (2005). Chunk limits and length limits in immediate recall: a reconciliation. J. Exp. Psychol. Learn. Mem. Cogn. 31, 1235–1249 10.1037/0278-7393.31.6.1235 16393043 PMC2673719

[B15] Cohen N. J. Eichenbaum H. (1993). Memory, Amnesia, and the Hippocampal System. Cambridge, MA: MIT Press

[B16] Cowan N. (2001). The magical number 4 in short-term memory: a reconsideration of mental storage capacity. Behav. Brain Sci. 24, 87–185 10.1017/S0140525X01003922 11515286

[B17] Cowan N. (2011). The focus of attention as observed in visual working memory tasks: making sense of competing claims. Neuropsychologia 49, 1401–1406 10.1016/j.neuropsychologia.2011.01.035 21277880 PMC3095706

[B18] Cowan N. Chen Z. Rouder J. N. (2004). Constant capacity in an immediate serial-recall task: a logical sequel to Miller (1956). Psychol. Sci. 15, 634–640 10.1111/j.0956-7976.2004.00732.x 15327636

[B19] Cowan N. Li D. Moffitt A. Becker T. M. Martin E. A. Saults J. S. (2011). A neural region of abstract working memory. J. Cogn. Neurosci. 23, 2852–2863 10.1162/jocn.2011.21625 21261453 PMC3138911

[B20] Dahlin E. Stigsdotter-Neely A. Larsson A. Bäckman L. Nyberg L. (2008). Transfer of learning after updating training mediated by the striatum. Science 320, 1510–1512 10.1126/science.1155466 18556560

[B21] Eichenbaum H. Yonelinas A. R. Ranganath C. (2007). The medial temporal lobe and recognition memory. Annu. Rev. Neurosci. 30, 123–152 10.1146/annurev.neuro.30.051606.094328 17417939 PMC2064941

[B22] Ericsson K. (2003). Exceptional memorizers: made, not born. Trends Cogn. Sci. 7, 233–235 10.1016/S1364-6613(03)00103-7 12804685

[B23] Ericsson K. A. Kintsch W. (1995). Long-term working memory. Psychol. Rev. 102, 211–245 10.1037/0033-295X.102.2.211 7740089

[B24] Ericsson K. A. Kintsch W. (2000). Shortcomings of generic retrieval structures with slots of the type of Gobet (1993) proposed and modeled. Br. J. Psychol. 91, 571–590 10.1348/000712600161998 11104179

[B25] Frey P. W. Adesman P. (1976). Recall memory for visually presented chess positions. Mem. Cognit. 4, 541–547 10.3758/BF03213216 21286979

[B26] Gabrieli J. D. Brewer J. B. Desmond J. E. Glover G. H. (1997). Separate neural bases of two fundamental memory processes in the human medial temporal lobe. Science 276, 264–266 10.1126/science.276.5310.264 9092477

[B27] Glabus M. F. Horwitz B. Holt J. L. Kohn P. D. Gerton B. K. Callicott J. H. (2003). Interindividual differences in functional interactions among prefrontal, parietal and parahippocampal regions during working memory. Cereb. Cortex 13, 1352–1361 10.1093/cercor/bhg082 14615300

[B28] Glanzer M. Fisher B. Dorfman D. (1984). Short-term storage in reading. J. Verbal Learn. Verbal Behav. 23, 467–486 10.1016/S0022-5371(84)90300-1

[B29] Gobet F. (2000a). Some shortcomings of long-term working memory. Br. J. Psychol. 91, 551–570 10.1348/000712600161989 11104178

[B30] Gobet F. (2000b). Retrieval structures and schemata: a brief reply to Ericsson and Kintsch. Br. J. Psychol. 91, 591–594 10.1348/000712600162005 11104179

[B31] Gobet F. Clarkson G. (2004). Chunks in expert memory: evidence for the magical number four … or is it two. Memory 12, 732–747 10.1080/09658210344000530 15724362

[B32] Gobet F. Simon H. A. (1996). Templates in chess memory: a mechanism for recalling several boards. Cogn. Psychol. 31, 1–40 10.1006/cogp.1996.0011 8812020

[B33] Guida A. Gobet F. Tardieu H. Nicolas S. (2012). How chunks, long-term working memory and templates offer a cognitive explanation for neuroimaging data on expertise acquisition: a two-stage framework. Brain Cogn. 79, 221–244 10.1016/j.bandc.2012.01.010 22546731

[B34] Guida A. Gras D. Noel Y. Le Bohec O. Quaireau C. Nicolas S. (2013). The effect of long-term working memory through personalization applied to free recall: uncurbing the primacy effect enthusiasm. Mem. Cognit. 41, 571–587 10.3758/s13421-012-0284-3 23297048

[B35] Guida A. Tardieu H. Nicolas S. (2009). The personalisation method applied to a working memory task: evidence of long-term working memory effects. Eur. J. Cogn. Psychol. 21, 862–896 10.1080/09541440802236369

[B36] Hill N. M. Schneider W. (2006). Brain changes in the development of expertise: Neurological evidence on skill-based adaptations, in Cambridge Handbook of Expertise and Expert Performance, eds Ericsson K. A. Charness N. Feltovich P. Hoffman R. (New York, NY: Cambridge University), 653–682 10.1017/CBO9780511816796.037

[B37] Jeneson A. Wixted J. T. Hopkins R. O. Squire L. R. (2012). Visual working memory capacity and the medial temporal lobe. J. Neurosci. 32, 3584–3589 10.1523/JNEUROSCI.6444-11.2012 22399780 PMC3349278

[B37a] Jonides J. Lewis R. L. Nee D. E. Lustig C. A. Berman M. G. Moore K. S. (2008). The mind and brain of short-term memory. Annu. Rev. Psychol. 59, 193–224 10.1146/annurev.psych.59.103006.093615 17854286 PMC3971378

[B38] Kelly A. M. C. Garavan H. (2005). Human functional neuroimaging of brain changes associated with practice. Cereb. Cortex 15, 1089–1102 10.1093/cercor/bhi005 15616134

[B39] Kondo Y. Suzuki M. Mugikura S. Abe N. Takahashi S. Iijima T. (2005). Changes in brain activation associated with use of a memory strategy: a functional MRI study. Neuroimage 24, 1154–1163 10.1016/j.neuroimage.2004.10.033 15670693

[B40] Lee A. C. H. Rudebeck S. R. (2010). Investigating the interaction between spatial perception and working memory in the human medial temporal lobe. J. Cogn. Neurosci. 22, 2823–2835 10.1162/jocn.2009.21396 19925184 PMC2929461

[B41] Lepage M. Habib R. Tulving E. (1998). Hippocampal PET activations of memory encoding and retrieval: the HIPER model. Hippocampus 8, 313–322 10.1002/(SICI)1098-1063(1998)8:4<313::AID-HIPO1>3.0.CO;2-I 9744418

[B42] McIntosh A. R. (1998). Understanding neural interactions in learning and memory using functional neuroimaging. Ann. N.Y. Acad. Sci. 85, 556–571 10.1111/j.1749-6632.1998.tb10625.x 9929651

[B43] Majerus S. D'Argembeau A. Perez T. M. Belayachi S. Van der Linden M. Collette F. (2010). The commonality of neural networks for verbal and visual short-term memory. J. Cogn. Neurosci. 22, 2570–2593 10.1162/jocn.2009.21378 19925207

[B44] Mathy F. Feldman J. (2012). What's magic about magic numbers. Chunking and data compression in short-term memory. Cognition 122, 346–362 10.1016/j.cognition.2011.11.003 22176752

[B45] Miller G. A. (1956). The magical number seven, plus or minus two: some limits of our capacity for processing information. Psychol. Rev. 63, 81–97 10.1037/h0043158 13310704

[B46] Oakes L. M. Ross-Sheehy S. Luck S. J. (2006). Rapid development of feature binding in visual short-term memory. Psychol. Sci. 17, 781–787 10.1111/j.1467-9280.2006.01782.x 16984295

[B47] Olson I. R. Moore K. Stark M. Chatterjee A. (2006). Visual working memory is impaired when the medial temporal lobe is damaged. J. Cogn. Neurosci. 18, 1087–1097 10.1162/jocn.2006.18.7.1087 16839283

[B48] Pesenti M. Zago L. Crivello F. Mellet E. Samson D. Duroux B. (2001). Mental calculation in a prodigy is sustained by right prefrontal and medial temporal areas. Nat. Neurosci. 4, 103–107 10.1038/82831 11135652

[B50] Prabhakaran V. Narayanan K. Zhao Z. Gabrieli J. D. E. (2000). Integration of diverse information in working memory within the frontal lobe. Nat. Neurosci. 3, 85–90 10.1038/71156 10607400

[B51] Raffone A. Wolters G. (2001). A cortical mechanism for binding in visual working memory. J. Cogn. Neurosci. 13, 766–785 10.1162/08989290152541430 11564321

[B52] Ranganath C. Blumenfeld R. S. (2005). Doubts about double dissociations between short- and long-term memory. Trends Cogn. Sci. 9, 374–380 10.1016/j.tics.2005.06.009 16002324

[B53] Reichle E. D. Carpenter P. A. Just M. A. (2000). The neural bases of strategy and skill in sentence-picture verification. Cogn. Psychol. 40, 261–295 10.1006/cogp.2000.0733 10888341

[B54] Scalf P. E. Banich M. T. Kramer A. F. Narechania K. Simon C. D. (2007). Double take: parallel processing by the cerebral hemispheres reduces attentional blink. J. Exp. Psychol. Hum. Percept. Perform. 33, 298–329 10.1037/0096-1523.33.2.298 17469970

[B55] Shafritz K. M. Gore J. C. Marois R. (2002). The role of the parietal cortex in visual feature binding. Proc. Natl. Acad. Sci. U.S.A. 99, 10917–10922 10.1073/pnas.152694799 12149449 PMC125073

[B56] Shrager Y. Levy D. A. Hopkins R. O. Squire L. R. (2008). Working memory and the organization of brain systems. J. Neurosci. 28, 4818–4822 10.1523/JNEUROSCI.0710-08.2008 18448658 PMC2426968

[B57] Squire L. R. Stark C. E. L. Clark R. E. (2004). The medial temporal lobe. Annu. Rev. Neurosci. 27, 279–306 10.1146/annurev.neuro.27.070203.144130 15217334

[B58] Squire L. R. Wixted J. (2011). The cognitive neuroscience of human memory since H.M. Annu. Rev. Neurosci. 34, 259–288 10.1146/annurev-neuro-061010-113720 21456960 PMC3192650

[B59] Todd J. J. Marois R. (2004). Capacity limit of visual short-term memory in human posterior parietal cortex. Nature 428, 751–754 10.1038/nature02466 15085133

[B60] Vogel E. K. Machizawa M. G. (2004). Neural activity predicts individual differences in visual working memory capacity. Nature 428, 748–751 10.1038/nature02447 15085132

[B61] Young B. J. Otto T. Fox G. D. Eichenbaum H. (1997). Memory representation within the parahippocampal region. J. Neurosci. 17, 5183–5195 9185556 10.1523/JNEUROSCI.17-13-05183.1997 PMC6573311

